# VIBRA trial – Effect of village-based refill of ART following home-based same-day ART initiation vs clinic-based ART refill on viral suppression among individuals living with HIV: protocol of a cluster-randomized clinical trial in rural Lesotho

**DOI:** 10.1186/s13063-019-3510-5

**Published:** 2019-08-22

**Authors:** Alain Amstutz, Thabo Ishmael Lejone, Lefu Khesa, Josephine Muhairwe, Bienvenu Lengo Nsakala, Katleho Tlali, Moniek Bresser, Fabrizio Tediosi, Mathebe Kopo, Mpho Kao, Thomas Klimkait, Manuel Battegay, Tracy Renée Glass, Niklaus Daniel Labhardt

**Affiliations:** 1 Clinical Research Unit, Department of Medicine, Swiss Tropical and Public Health Institute, Socinstrasse 57, 4051 Basel, Switzerland; 2 University of Basel, 4051 Basel, Switzerland; 3 Division of Infectious Diseases and Hospital Epidemiology, University Hospital Basel, 4051 Basel, Switzerland; 4 SolidarMed, Swiss Organization for Health in Africa, Butha-Buthe, Lesotho; 5 Butha-Buthe Government Hospital, Butha-Buthe, Lesotho; 6 Molecular Virology, Department of Biomedicine, University of Basel, 4051 Basel, Switzerland

**Keywords:** HIV, cluster randomized controlled trial, village health worker, community health worker, home-based, differentiated care and delivery, antiretroviral therapy, Lesotho, Southern Africa, multi component intervention

## Abstract

**Background:**

There is a need for evaluating community-based antiretroviral therapy (ART) delivery models to improve overall performance of HIV programs, specifically in populations that may have difficulties to access continuous care. This cluster-randomized clinical trial aims to evaluate the effectiveness of a multicomponent differentiated ART delivery model (VIBRA model) after home-based same-day ART initiation in remote villages in Lesotho, southern Africa.

**Methods/design:**

The VIBRA trial (VIllage-Based Refill of ART) is a cluster-randomized parallel-group superiority clinical trial conducted in two districts in Lesotho, southern Africa. Clusters (i.e., villages) are randomly assigned to either the VIBRA model or standard care. The clusters are stratified by district, village size, and village access to the nearest health facility. Eligible individuals (HIV-positive, aged 10 years or older, and not taking ART) identified during community-based HIV testing campaigns are offered same-day home-based ART initiation. The intervention clusters offer a differentiated ART delivery package with two features: (1) drug refills and follow-ups by trained and supervised village health workers (VHWs) and (2) the option of receiving individually tailored adherence reminders and notifications of viral load results via SMS. The control clusters will continue to receive standard care, i.e., collecting ART refills from a clinic and no SMS notifications. The primary endpoint is viral suppression 12 months after enrolment. Secondary endpoints include linkage to and engagement in care. Furthermore, safety and cost-effectiveness analyses plus qualitative research are planned. The minimum target sample size is 262 participants. The statistical analyses will follow the CONSORT guidelines. The VIBRA trial is linked to another trial, the HOSENG (HOme-based SElf-testiNG) trial, both of which are within the GET ON (GETing tOwards Ninety) research project.

**Discussion:**

The VIBRA trial is among the first to evaluate the delivery of ART by VHWs immediately after ART initiation. It assesses the entire HIV care cascade from testing to viral suppression. As most countries in sub-Saharan Africa have cadres like the VHW program in Lesotho, this model—if shown to be effective—has the potential to be scaled up. The system impact evaluation will provide valuable cost estimations, and the qualitative research will suggest how the model could be further modified to optimize its impact.

**Trial registration:**

Clinicaltrials.gov, NCT03630549. Registered on 15 August 2018.

**Electronic supplementary material:**

The online version of this article (10.1186/s13063-019-3510-5) contains supplementary material, which is available to authorized users.

## Introduction

Multiple studies conducted in sub-Saharan Africa (SSA) report high attrition from HIV testing to linkage to care and suboptimal engagement in care [1–7]. There are many reasons for this, but structural barriers such as the time-consuming and expensive (pre-)antiretroviral therapy (ART) visits and subsequent regular drug refill visits represent major impediments, especially in rural settings [8–16]. Therefore, the World Health Organization (WHO), international funders, national policies in sub-Saharan Africa, and the research community are calling for differentiated ART delivery models that are adapted to the local context. These include further shifting of tasks and the decentralization of care [17–23].

The CASCADE trial was a randomized clinical trial on the effect of offering same-day ART vs the usual referral to a health facility during home-based HIV testing on linkage to care and viral suppression among adults with HIV in Lesotho. This trial evaluated same-day ART initiation in the community and found significantly improved outcomes along the entire HIV care cascade [24]. However, the CASCADE trial did not quite reach the targeted 90% linkage to and engagement in care rates after participants had tested positive for HIV and were offered same-day ART initiation at home. The VIBRA (Village-Based Refill of ART) trial builds on these findings, specifically addressing the challenges after same-day home-based ART initiation.

For patients who are stable on ART, decentralization of care to the community level and the shifting of care tasks to lay health workers has been shown to be feasible, cost-effective, and acceptable [25–40]. The WHO, thus, endorses the recruitment of community health workers as a strategy to mitigate the impact of the severe shortage of nurses and doctors in rural Africa on healthcare coverage [41]. Moreover, the United Nations Program on HIV/AIDS (UNAIDS) launched a plan to recruit 2 million community health workers in Africa to support its strategy [42]. Lesotho, a small landlocked country surrounded by South Africa, has the second-highest adult HIV prevalence globally (25.6%). More than 70% of its population live in rural areas where there is a shortage of doctors and nurses [43, 44]. A long-standing public sector cadre of lay personnel, called village health workers (VHWs), was introduced in 1978 and more than 4000 VHWs are currently successfully operating in all districts of Lesotho [45].

We designed the VIBRA model in close collaboration with local stakeholders. It is a multicomponent differentiated ART delivery package that builds on the VHW program and SMS technology. In this manuscript, we describe the protocol for a cluster-randomized clinical trial that aims to evaluate the effectiveness of the VIBRA model following same-day home-based ART initiation in rural communities in Lesotho.

## Methods/design

### Setting

The VIBRA trial will be conducted in the districts of Butha-Buthe and Mokhotlong in northern Lesotho, in the catchment areas of 22 health facilities. Both districts are mostly rural with an estimated population of 220,000, mainly living in villages scattered over a mountainous area of 5842 km^2^. The trial utilizes the long-standing VHW country program. VHWs are members of and appointed by the community to provide a package of basic services at the household level, although they have no formal professional health education. They are elected by the village members, complete 2 weeks of training followed by periodic refresher courses, and are supported and supervised by the health center staff for the corresponding catchment area. Most are supported by the Ministry of Health and receive a monthly stipend of USD 20.

### Design

The VIBRA trial is a cluster-randomized superiority trial. The trial is linked to another trial, the HOSENG (HOme-based SElf-testiNG) trial [76]. Together, HOSENG and VIBRA constitute the GET ON (GETing tOwards Ninety) research project. Reasons for this interlinked design are that both trials rely on interventions involving VHWs, who need to be randomized and specifically trained, and that the HOSENG trial provides one of the recruitment platforms for VIBRA. Thus, the two trials are based on the same cluster randomization and will run in parallel. This design allows us to assess the entire HIV care cascade in one larger project. A cluster-randomized design was chosen because of the reliance on VHWs and the high risk of cross-contamination between study arms if randomization were done at the individual level.

### Cluster randomization, screening of study participants, eligibility and interventions

Cluster eligibility, cluster sampling, cluster randomization, and the HIV testing campaign are described in detail in the interlinked HOSENG study protocol, which has been published separately [76]. In short, before the trial started, the eligible clusters (i.e., villages) were randomized into four groups: (1) VIBRA control and HOSENG control, (2) VIBRA control and HOSENG intervention, (3) VIBRA intervention and HOSENG control, and (4) VIBRA intervention and HOSENG intervention. The randomization is stratified by district, village size (≥30 vs < 30 households) and access to the nearest health facility (easy vs hard to reach, defined by needing to cross a mountain or river, or > 10 km away from the health facility), in a 1:1:1:1 allocation ratio with block sizes of 4. In total, 159 clusters were identified and randomized into one of the four groups. When enrolment started, 25 clusters per group, i.e., 50 clusters per arm (VIBRA intervention vs control) were available to the local study team with the option to add more clusters as needed to reach the recruitment goals.

Campaign teams consisting of counsellors and one study nurse visit the rural villages (clusters) in the two study districts. The teams offer HIV testing and counselling and multi-disease screening and prevention. Household members who are eligible for and consent, undergo HIV testing by the counselors according to national HIV testing guidelines [46]. All household members with a confirmed HIV-positive result and not taking ART are screened by the study nurse for VIBRA eligibility according to the criteria in Table 1.

**Table 1:** Eligibility criteria for VIBRA trial

Inclusion criteria	Exclusion criteria
Individual is a member of a visited household, i.e. the individual is (a) acknowledged by the household head or their representative as part of the household and (b) sleeps in the household regularly (at least once a month)	HIV-positive individual is on ART or stopped less than 30 days ago
Individual is confirmed HIV-positive, as determined by two reactive blood-based HIV antibody tests according to national guidelines	HIV-positive individual is physically, mentally, or emotionally not able to participate in the study, in the opinion of the study nurse
Individual has never taken ART (ART naïve) or has stopped ART more than 30 days prior (ART defaulter according to national guidelines)	HIV-positive individual is in care for hypertension or diabetes; documentation or proof of medication is needed
Individual is ≥10 years old and body weight ≥ 35 kg	HIV-positive individual wishes to get care outside the two study districts

*ART* antiretroviral therapy, *HIV* human immunodeficiency virus

If a patient is eligible for VIBRA and consents, the study nurse offers same-day home-based ART initiation and proposes follow-up care according to the cluster assignment. As successfully implemented through our previous trial and recommended by the national guidelines, same-day home-based ART initiation, using the national standard first-line ART regimen, will be provided in both arms [19, 24]. If individuals are not eligible for the VIBRA trial and thus, not eligible for same-day standard first-line ART initiation, they are referred to the health facility. Features of same-day ART initiation are outlined in Table 2.

**Table 2:** Components of same-day ART initiation in the VIBRA trial

Component	Description	Remarks
Medical history	The study nurse assesses the patient’s medical history using a checklist; if necessary the nurse may refer the patient to a health facilty and may choose not to initiate same-day ART. In case of doubt, the nurse will contact the study physician	See checklist in Additional file 1 (eForm title “Medical history”)
Physical examination	The study nurse conducts a structured physical examination using a checklist; if necessary the nurse may refer the patient to a health facilty and may choose not to initiate same-day ART. In case of doubt, the nurse will contact the study physician	See checklist in Additional file 1 (eForm title “Physical exam”)
WHO stage	The study nurse assigns a clinical WHO stage according to the physical examination and medical history	
CD4 measurement	The study team performs a point-of-care CD4 count, using PIMA Alere™ (a finger-prick test), which gives results within 20 min. ▪ If CD4 count < 350 cells/mcL: co-trimoxazole prophylaxis, 960 mg once daily orally, 1 tablet ◦ If participant < 14 years: 1/2 tablet once daily orally ▪ If CD4 count < 200 cells/mcL: cryptococcal antigen (CrAg) point-of-care measurement (lateral flow assay, IMMY©) ◦ If CrAg positive: immediate referral to a nearby district hospital by the study team and no same-day ART initiation	Although baseline CD4 counts are no longer used according to national guidelines to establish ART eligibility, the baseline CD4 count remains a strong indicator of early outcomes on ART and is, therefore, (a) an important variable for the study analysis and (b) an important clinical monitoring measurement for the prevention of opportunistic infections The national guidelines suggest screening for CrAg only if CD4 count < 100 cells/mcL. However, data are scarce, so we will extend screening to those with CD4 count < 200 cells/mcL For CrAg-positive patients, preventive or therapeutic antifungal treatment is indicated and a lumbar puncture is required. Thus, referral to the hospital is needed. Due to evidence that early ART initiation should be avoided if there is cryptococcal meningitis, same-day ART will not be initiated in CrAg-positive individuals until cryptococcal meningitis has been excluded
Creatinine measurement	The study team performs a point-of-care creatinine measurement, using StatSensor Creat™ Nova™ Biomedical (finger-prick test), which gives results within 2 min. • If estimated creatinine glomerular filtration rate according to the Cockroft–Gault equation < 50 mL/min: substitution of tenofovir disoproxil fumarate (TDF) with abacavir or zidovudine depending on the hemoglobin result • If estimated creatinine glomerular < 30 mL/min: the nurse may refer the patient to a health facility and may choose not to initiate same-day ART	According to national guidelines, a baseline creatinine measurement is needed before initiating standard first-line ART including TDF
Hemoglobin measurement	The study nurse performs a point-of-care hemoglobin measurement, using Hemocue^TM^, HB301 (a finger-prick test), which gives results within 2 min. • If hemoglobin < 8 g/dL: zidovudine is contraindicated and the nurse may refer the patient to a health facility and may choose not to initiate same-day ART	According to national guidelines, hemoglobin must be measured before initiating an ART regimen containing zidovudine
Adherence counseling and education session	The study nurse conducts a structured education and adherence session, which is delivered using a leaflet, one-on-one, in approximately 5–10 min.	A condensed version of the education and counseling typically provided over the course of the former pre-ART visits was developed for and successfully tested in the previous trial (CASCADE trial)
Readiness assessment	Before dispensing ART, the study nurse will confirm the patient’s readiness and will answer all remaining questions, using a checklist • If the patient is not ready, they are referred to a health facility and same-day ART is not initiated	See checklist in Additional file 1 (eForm title “Readiness”)
Dispensing of ART	The study nurse prescribes a 1-month supply of the standard first-line ART according to national guidelines: TDF/lamivudine/efavirenz as a fixed-dose combination, once daily • If TDF is contraindicated, it is substituted with abacavir or zidovudine depending on the hemoglobin level • If the patient has an uncontrolled mental disease (e.g., active psychosis), they are referred to a health facility and same-day ART is not initiated • Depending on CD4 count, additionally 1 month’s supply of co-trimoxazole will be dispensed	The study nurses, like other qualified nurses in Lesotho, are authorized to write prescriptions for ART. We include only patients aged 10 years and older and weighing 35 kg or above (see the eligibility criteria). Thus, TDF/lamivudine/efavirenz is the standard treatment, which everybody will receive unless we discover they have renal impairment Before dispensing ART, the study nurse will re-test the patient again for HIV as per national ART guidelines
Follow-up visit	The study nurse provides a follow-up date in 12–16 days, either at the health facility (VIBRA control) or with the VHW (VIBRA intervention, if the participant agrees), as their next ART visit	The study nurse documents the entire process in the patient booklet (“bukana”), including drugs prescribed and follow-up date, and fills in all government documents (patient file and registers) at the health facility responsible for the catchment area

*ART* antiretroviral therapy, *CrAg* cryptococcal antigen, *HIV* human immunodeficiency virus, *TDF* tenofovir disoproxil fumarate, *VHW* village health worker, *WHO* World Health Organization

#### Intervention clusters

**Fig. 1:**
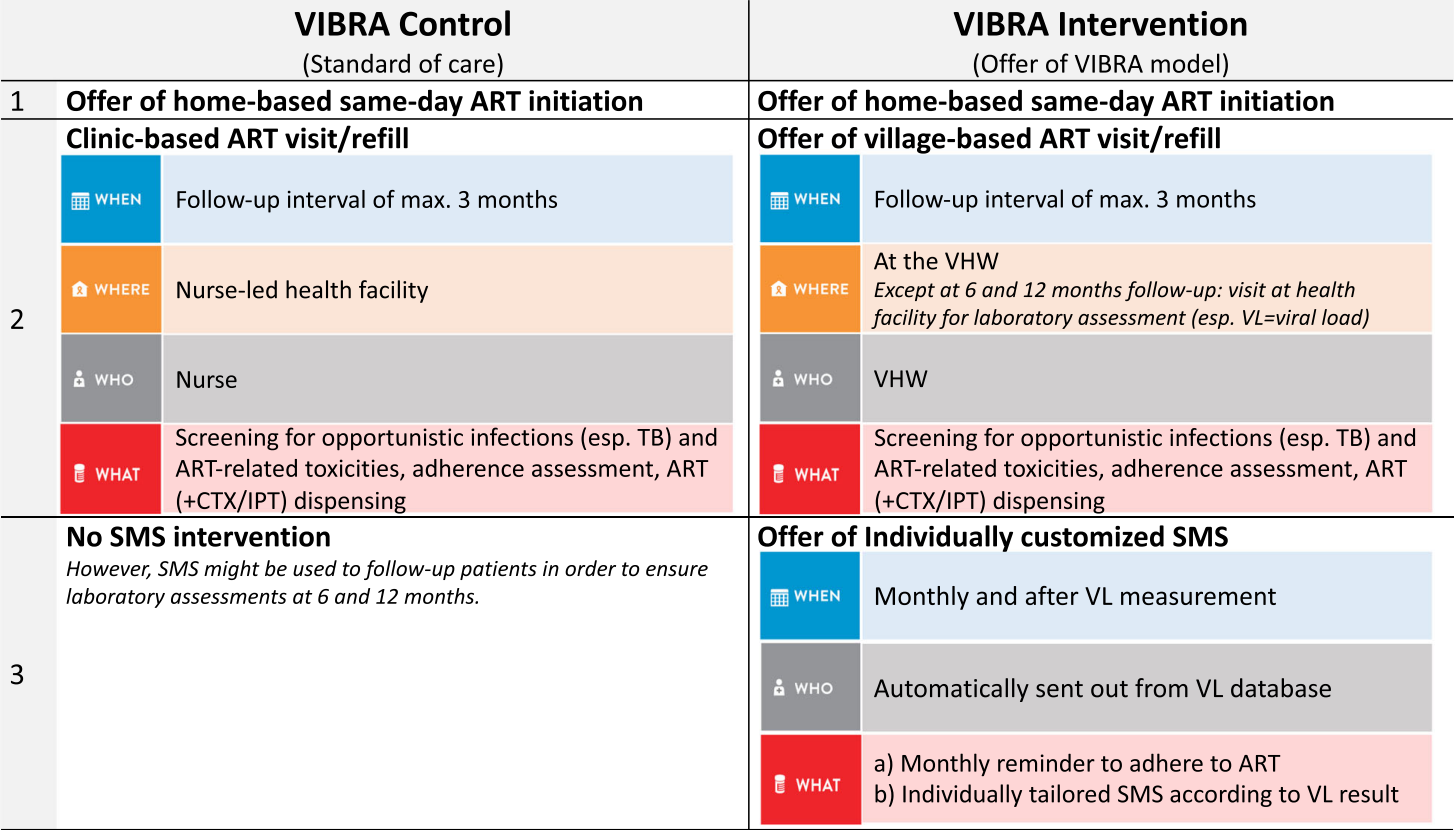
Procedures for the VIBRA intervention and control clusters

The participants in the intervention clusters are offered the two features of the VIBRA model. The first feature is the possibility of village-based ART visits and refills through the VHWs, with routine clinic visits required only at 6 and 12 months after ART initiation. The second is the offer of receiving a tailored SMS intervention. Figure 1 summarizes the VIBRA model.

If participants in the intervention clusters choose the village-based ART visits and refills, they are given an appointment for a first clinical visit to the VHW 12 to 16 days after the home-based ART initiation. At each visit, the VHW follows the same paper-based checklist (provided as Additional file 1: eForm title “Follow-up”) written in the local language (Sesotho). By following the checklist, the VHW documents: (a) the patient’s symptoms to alert them to potential drug toxicity, opportunistic infections, or immune reconstitution inflammatory syndrome, (b) adherence to ART, and (c) any visits to other health facilities. To ensure safe and high-quality clinical management, participants in intervention clusters will not only be linked to their VHW, but will also be under the care of the community ART nurse (CAN) for the relevant district. If any question on the checklist triggers an alert, the VHW will inform their CAN. CANs are nurses who are experienced in HIV care. One CAN per district has been hired. The VHWs and CANs will have a list of the participants for whom they are responsible. A VHW will be allowed to dispense ART only to participants on their list. Like the health facilites, the VHW will provide a supply of drugs for 1–3 months at each visit. Participants are, however, encouraged to visit the VHW or the clinic at any time when problems or questions arise. Six months after ART initiation, the participant must attend the clinic for the first time for a laboratory assessment.

VHWs have monthly meetings at the health facility with a designated facility staff member. The VIBRA model will utilize these existing meetings and the CAN (or a representative) will join and provide support. These meetings are a platform for reviewing patient files. Patients can be up-referred (to the health facility) or down-referred (to the VHW). If a patient misses an ART visit, they will be traced by the VHW using a standardized tracking tool (provided as Additional file 1: eForm title “Tracing form”).

All VHWs in the intervention clusters will be trained to deliver the VIBRA model: (a) to dispense ART (and other co-medications such as cotrimoxazole), (b) to screen for ART-related adverse events and drug toxicities, (c) to screen for co-infection (especially tuberculosis), (d) to assess adherence, (e) to understand the referral algorithm in case there is clinical deterioration, (f) to address disclosure and keep confidentiality, and (g) to perform basic data entry for the checklists. This training will last for 2–3 days.

If participants in the intervention clusters choose the SMS intervention, they will receive monthly reminder SMS in Sesotho to adhere to ART (“Take your medication regularly as prescribed and don’t run out of medication”) and a viral load (VL) result-triggered SMS after the 6- and 12-month follow-up visits:

- If undetectable VL (< 20 copies/mL): “Congratulations, your lab test was good. Keep it up!”
- If detectable VL (≥20 copies/mL): “Your lab test results are back. Make sure to come to the health facility as soon as possible and remind the nurse about your lab test.”
- If technical failure of VL measurement: “The lab test was unsuccessful. Make sure to come to the health facility as soon as possible and remind the nurse about your lab test.”

To maintain participant confidentiality, messages will not explicitly mention HIV or HIV care. Participants are not asked to confirm receipt of messages or to reply and can choose at any time to opt out from receiving messages.

#### Control clusters

Participants in the control clusters are offered standard care, i.e., ART visits and refills at the health facility and no SMS intervention. They receive an appointment for a first clinic visit within 12 to 16 days of the home-based ART initiation. For each visit, a staff member at the health facility will fill in the same checklist as the VHWs. Study participants will not be offered any other differentiated delivery models.

### Endpoints

The primary endpoint is viral suppression (< 20 copies/mL) at 12 months, defined as the proportion of all participants with a suppressed VL 12 months (range: 10–15 months) after enrollment. Although this is a cluster-randomized trial, the analysis will be at individual level with viral suppression as a binary outcome. VL will be measured in plasma using the COBAS TaqMan® HIV-1 Test, v2.0 (Roche Diagnostics). Secondary and exploratory endpoints as well as the long-term follow-up are outlined in Table 3.

**Table 3:** Secondary and exploratory endpoints of the VIBRA trial

	Definition	Time point following enrolment	Remarks
Secondary endpoints			
Viral suppression < 20 copies/mL	Proportion of all participants with viral suppression (< 20 copies/mL)	6 (range: 5–8) months	
Viral suppression < 1000 copies/mL	Proportion of all participants with viral suppression (< 1000 copies/mL)	6 (range: 5–8) and 12 (range: 10–15) months	Some of the remote health facilities in our study districts face regular challenges in sending blood samples to a government hospital. To ensure there are sufficient VL measurements among our study participants, these health facilities will be equipped with dried-blood-spot kits as a backup for VL measurements. According to WHO, the recommended threshold for treatment failure using a dried blood spot is 1000 copies/mL
Linkage to care	Proportion of all participants attending their first clinic- or VHW-based ART visit at least once within the given period	a) Within 1 month b) Within 3 months	
Engagement in and retention in care	Proportion of all participants active in care at a health facility or with a VHW	6 (range: 5–8) and 12 (range: 10–15) months	Active in care is defined as at least one ART visit in the defined window. Patients who have stopped ART and those who have transferred to another health facility with a known outcome (documented proof of a follow-up visit or laboratory test) are included. Participants who have died, are lost to follow-up, who have transferred to another facility without a known outcome (no documented proof of a follow-up visit or laboratory test), or are more than 2 months late for a scheduled consultation or medication pick-up with a reason (e.g. currently no money for a clinic visit, busy working in South Africa, etc.) are not counted as being active in care
All-cause mortality	Proportion of participants dead for any reason	12 (range: 10–15) months	Verbal autopsy to determine cause of death whenever possible; death certificates and autopsy reports are not required
Lost to follow-up	Proportion of all participants lost to follow-up	12 (range: 10–15) months	We define participants lost to follow-up if they or their treatment buddies are more than 2 months late for a scheduled consultation or medication pick-up and we have no recent information about the participant
Transfer out	Proportion of all participants who transferred to another health facility (other than the one they were initially attached to) with a known outcome	12 (range: 10–15) months	As above, a known outcome is documented proof of a follow-up visit or laboratory test at the new health facility
Serious adverse event	Proportion of patients with a serious adverse event	Within 12 months	Serious adverse events graded according to the Division of AIDS Table for Grading the Severity of Adult and Pediatric Adverse Events, Version 2.0, November 2014
Exploratory endpoints			
Compliance with the protocol procedure	Proportion of ART refills and ART visits per participant according to the protocol schedule, at the VHW and the health facility	12 (range: 10–15) months	
Overall effect of HOSENG + VIBRA	Overall effect of the combined interventions HOSENG and VIBRA (arm 4 vs arm 1) on viral suppression (< 20 copies/mL)	12 (range: 10–15) months	
Assessment of acceptance of interventions	a) Acceptance of same-day ART initiation b) Acceptance of VIBRA model	Within 1 month	
Long-term follow-up			
Long-term follow-up	Proportion of participants who are active in care and virologically suppressed (< 20 copies/mL)	24 (range: 22–28) months	

The denominator of all proportions is the total number of study participants enrolled. Although this is a cluster-randomized trial, these endpoints will be analyzed at the individual level with binary outcomes

*ART* antiretroviral therapy, *VHW* village health worker, *VL* viral load

### Additional research within the project

We will conduct biomolecular research within this project. We will assess the prevalence of major drug resistance mutations in baseline samples and on all samples with unsuppressed VL at 12 months. Participants who start ART at home during the testing campaign but subsequently never link to care will be specifically traced to assess the development of drug resistance mutations.

Qualitative research is planned alongside the project to provide important contextual data and an in-depth exploration of the community response to the intervention. For a qualitative case–control study, a random sample from the VIBRA intervention clusters will be chosen. Cases will be participants who refuse village-based ART refills through the VHW, while controls will be participants who accept village-based ART refills through the VHW. Moreover, we will conduct standardized interviews with a random sample of all stakeholders involved in delivering this new ART care and delivery model.

We will perform a system impact evaluation and cost-effectiveness analysis to estimate the impact of the VIBRA intervention on health benefits and costs. First, we will assess the direct costs of the interventions. Secondly, we will assess the cost-effectiveness of the VIBRA model. Thirdly, we will assess the economic burden of the interventions to the participants, i.e., including both direct costs and the opportunity costs of their time. The assessment of direct costs will include staff costs (campaign staff, clinical staff, laboratory staff, VHWs, and CANs), personnel training costs, the cost of equipment (costs of HIV tests, costs of ART and other drugs, and laboratory costs including the point-of-care tests at enrolment), and non-medical costs for the participant. The VIBRA model is expected to reduce the number of clinic visits because of the VHW-based ART refill. There are expected to be fewer unscheduled visits because the intervention may lead to a better and more sustained clinical outcome. This would decrease costs for the health system and the participants (i.e., time required to access care, lost working time while accessing care, and additional expenses while accessing care).

### Data collection and management, biologic material, and follow-ups

The VHWs and the healthcare staff at the health facilities will collect data from scheduled and unscheduled ART visits on standardized paper study forms (case report forms), which are the source documents. Case report forms will be collected regularly by the study team and entered into a password-protected database (MACRO, Elsevier). Similarly, data relevant for the SMS intervention will be entered and stored in a separate encrypted and password-protected online database. This offers the possibility of sending out SMS messages automatically and is connected to the district laboratory database containing the VL results. The platform and data are housed on a dedicated server in a data center in Switzerland (Interxion, managed by Hostpoint AG), which meets FINMA-RS 08/07 requirements, is ISO-27001-certified, and encrypts data in-transit with SSL and all patient names at-rest using OpenSSL with the AES-256-CTR cipher method. Access to both platforms is strictly limited and regulated through personal user profiles. SMS messages are dispatched using the trusted third-party provider Twilio, headquartered in the United States and certified with the EU–U.S. and Swiss–U.S. Privacy Shield Framework. Consent forms will be stored securely in the headquarters of the study center (the SolidarMed office in Butha-Buthe, Lesotho). Participant files will be maintained in storage for at least 10 years after completion of the study.

**Fig. 2:**
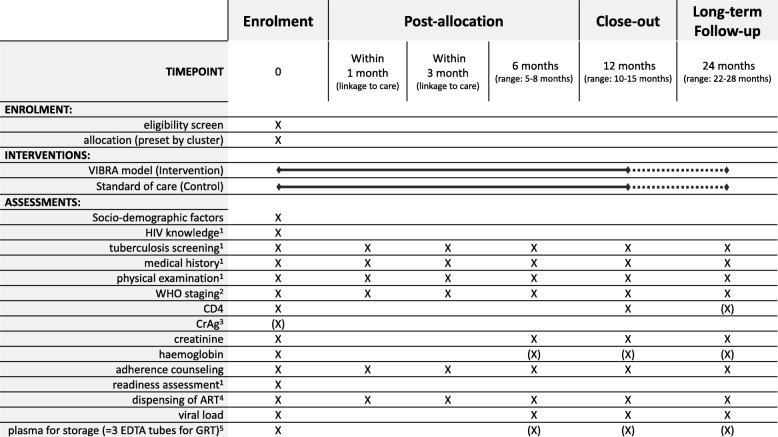
SPIRIT flow diagram for the VIBRA trial. Footnotes: ^1^see Additional file 1, ^2^only at clinic-based follow-up visits, ^3^for all participants with a baseline CD4-count <200 cells/mcL (see also Table 2), ^4^incl. CTX and IPT (+B6) and other co-infection (prophylaxis) medication if appropriate, ^5^See chapter Additional research within the project

Participants in all clusters undergo HIV testing and phlebotomy at enrolment, and phlebotomy at 6 and 12 months. For each participant, blood samples coded with the participant’s study ID will be stored at − 80 °C at the laboratory in Butha-Buthe Hospital. All samples collected fall under the biobank and material transfer agreement, which has been approved by the ethics committees in Switzerland and Lesotho. Figure 2 is the SPIRIT flow diagram and has an overview of data collection, laboratory assessments, and follow-up visits (Additional file 2).

### Sample size

Based on data from the CASCADE trial [24], we expect the proportion of patients engaged in care with documented viral suppression 12 months after same-day ART initiation in the control arm to be approximately 50% and we expect to recruit on average 4 individuals per cluster. Assuming a 20% refusal and ineligibility rate, about 400 individuals need to be screened to identify 320 eligible individuals and 90% power to detect a 20% increase in the intervention group. We plan to enroll a minimum of 262 patients to ensure a minimum power of 80%. Based on the assumption that we will recruit about 4 individuals per cluster, we will begin with 50 clusters per arm and can add more clusters as needed to reach our recruitment goals. All sample size calculations were done assuming a type 1 error rate of 0.05 and an intra-cluster correlation coefficient of 0.015. If the true number of eligible individuals per cluster is lower and thus more clusters will be needed to reach the targeted minimum sample size, this will result in an increase of power. Table 4 provides estimates of the sample size under varying recruitment scenarios.

**Table 4:** Sample size estimations for the VIBRA trial

Control rate of viral suppression	Intervention rate of viral suppression	Power	Intra-cluster correlation coefficient	Average number of eligible individuals per cluster	Total number of clusters	Total sample size
0.5	0.7	0.9	0.015	4	120	478
0.5	0.7	0.9	0.015	3	160	478
0.5	0.7	0.9	0.015	2	240	478
0.5	0.7	0.8	0.015	4	66	262
0.5	0.7	0.8	0.015	3	88	262
0.5	0.7	0.8	0.015	2	130	262
0.5	0.75	0.9	0.015	4	49	195
0.5	0.75	0.9	0.015	3	65	195
0.5	0.75	0.9	0.015	2	98	195
0.5	0.75	0.8	0.015	4	33	130
0.5	0.75	0.8	0.015	3	44	130
0.5	0.75	0.8	0.015	2	65	130

### Analyses

Analyses will be performed following the CONSORT guidelines for cluster-randomized trials [47] and an intention-to-treat principle including all participants as randomized per cluster randomization. Clusters are the unit of randomization, but individuals are the unit of analysis. As we expect to have many clusters (i.e., villages) with few eligible individuals (i.e., HIV-positive but not on ART), an individual-level analysis is most appropriate. Multi-level statistical models will be used to adjust for the clustered data. The following analysis sets will be used in this trial:

1. Intention-to-treat set: All study participants will be evaluated according to their cluster assignment at randomization.
2. Cluster per-protocol set: This set includes all participants from clusters that completed the study without a major protocol deviation.
3. Individual per-protocol set: This set includes all participants who completed the study without a major protocol deviation.

The primary analysis for the VIBRA study will be the comparison of viral suppression at 12 months after the offer of same-day ART initiation in the intention-to-treat set. The primary analysis will use a multi-level logistic regression model to assess the difference between the arms, adjusted for the prespecified randomization stratification factors and the clustering according to village. Moreover, we will adjust for the most important baseline characteristics if found to be unbalanced (gender, age, known HIV status vs newly diagnosed, ever on ART vs never on ART, CD4 count) and other factors found to be largely unbalanced between the intervention and control clusters.

Baseline characteristics will be presented according to randomized groups and no formal testing will be performed. Categorical variables will be described as absolute and relative frequencies and continuous variables as medians and interquartile ranges. As with the primary analysis, secondary endpoints will be analyzed with a multi-level logistic regression model. All results will be presented as odds ratios and their respective 95% confidence intervals. Several sensitivity analyses will be conducted. We will do a quadrature check of the model fit and if found to be unreliable, we will utilize generalized estimating equations. The effect of sociodemographic and clinical determinants (age groups, gender, education status, employment status, WHO stage, CD4 count, tuberculosis status, CAGE status, HIV/ART history, and HIV knowledge) on key study outcomes will be assessed by including interaction terms in the model. If an interaction term is found to be significant, effect estimates will be summarized descriptively by subgroup. As the study is not powered for these pre-planned subgroup analyses, these results will be considered exploratory. Where data are missing in important covariates, multiple imputation will be utilized and the results compared to models ignoring missing data.

All analyses will be done using Stata (version 14, Stata Corporation, Austin, Texas, USA), using two-sided *p* values and a significance level of 0.05.

### Monitoring, auditing, and data safety and monitoring board

At least one external monitoring visit will assess adherence to the approved trial protocol, and the accuracy of completed case report forms and the electronic dataset. The VIBRA trial is researching the implementation of a treatment. The safety profiles of all drugs used are well known, and the intervention does not include any new drugs. Moreover, there have been encouraging results from similar trials in Uganda, Kenya, and Tanzania [48–50]. Thus, major adverse effects on patients’ health from this intervention are not expected. Participants in the VIBRA model can opt to switch back to usual care or be referred to facility-based care at any time during the trial period. Therefore, we do not intend to establish a data safety and monitoring board. However, a separate, detailed safety monitoring plan has been developed to handle adverse events and serious adverse events in line with Swiss and Basotho ethics regulations. Adverse events and serious adverse events will be graded according to the Division of AIDS Table for Grading the Severity of Adult and Pediatric Adverse Events, Version 2.0., November 2014 [51] and managed according to the standard procedure for each study site following the national guidelines [19]. The study physicians are responsible for all safety procedures. If a participant reports an adverse event of grade 2 or higher at their last study visit, they will remain under observation by the study physicians, even after study termination, until the adverse event is resolved or stabilized.

## Discussion

Effective and differentiated strategies are needed to improve the HIV care cascade, especially in rural settings. Despite successful upscaling of ART, the financial, human, and physical resources available to fulfill the UNAIDS targets are unlikely to grow relative to the increasing number of people on ART [52, 53]. Therefore, there is global consensus that new differentiated care and service delivery models that increase the capacity, efficiency, and cost-effectiveness of delivering ART without reducing quality of care are urgently needed [22, 39, 54].

Shifting tasks to lay health workers in the community is a promising approach and in line with the current UNAIDS initiative [42]. However, at community level, task-shifting usually focuses on adherence monitoring, not the provision of antiretroviral drugs [29, 55–61]. A few programs use community health workers or VHWs to supply ART at home to patients [48, 49, 62–67]. This is, however, a resource-intensive intervention, there may be difficulties with confidentiality and stigma relating to home visits, patients must be at home during these visits, and their homes need to be readily accessible. These factors are unfavorable in a setting like Lesotho with limited resources and a population scattered around a vast mountainous area. Moreover, these models include only stable patients. The definition of a stable patient can be challenging, and may lead to late inclusion in these models. Differentiated care should be designed not only for stable patients but also for patients who would otherwise not engage in care [23].

The VIBRA model entails a second feature, SMS reminders and notifications. These have been studied widely in sub-Saharan Africa and lead to increased adherence and engagement in care [68–74]. However, sustainability is questionable, especially when reminders must be implemented manually. Our setting in Lesotho allows SMS messages to be sent automatically from an established database that is connected to the governmental district laboratory database with access to VL results from all study districts. Moreover, while most studies use standardized messages reminding the patient about their drug intake or clinic visits, the SMS intervention in the VIBRA model will go one step further. It will automatically generate and send notifications that are individually tailored according to the VL level, indicating the next steps of action for the patient.

This trial has several limitations. First, the study design does not allow for the evaluation of the effectiveness of each individual feature of the VIBRA model. Second, as in most operational research studies, we will have little control over what happens in our standard care arm. Standard care for HIV continues to evolve rapidly with frequent guideline revisions and the implementation of other differentiated ART service delivery models. Third, due to the nature of this pragmatic implementation trial, it is not possible to fully blind participants or staff to the intervention. However, allocation will be concealed from the study participants due to the cluster randomization design, which implies randomization before participant inclusion.

In summary, the VIBRA trial evaluates a unique differentiated ART delivery model with community-based drug refills and follow-ups after a home-based diagnosis and ART initiation in combination with a tailored SMS service. As most countries in sub-Saharan Africa have cadres like the VHW program in Lesotho, this model—if shown to be effective—has the potential to be scaled up. The system impact evaluation will provide valuable cost estimations, and the qualitative research will suggest how the model could be modified further to optimize its impact.

### Trial status and recruitment

The trial was launched on 16 August 2018 in both study districts. Based on the experience of previous HIV testing campaigns and the CASCADE trial, we assumed that we would reach the required minimum target sample size in about 6–8 months. The initial study protocol, version 5, was submitted to the ethics committees in Lesotho (February 2018) and Switzerland (April 2018), and approved on 25 April 2018 (Lesotho) and 8 May 2018 (Switzerland). Meanwhile, two minor amendments to the study protocol were submitted and have been accepted. The current protocol is version 7 and was approved in October 2018.

## Additional file

Additional file 1: GET ON electronic case report form. (PDF 1050 kb)

Additional file 2: SPIRIT 2013 Checklist: Recommended items to address in a clinical trial protocol and related documents. (PDF 82 kb)

## Data Availability

The datasets used and analyzed during the study will be available from the corresponding author on reasonable request.

## Abbreviations

ART: Antiretroviral therapy
CAN: Community ART nurse
CrAg: Cryptococcal antigen
GET ON: Getting Towards Ninety
HIV: Human immunodeficiency virus
HOSENG: Home-based Self-testing
SMS: Short Message Service
TDF: Tenofovir disoproxil fumarate
UNAIDS: United Nations Program on HIV/AIDS
VHW: Village health worker
VIBRA: Village-based Refill of ART
VL: Viral load
WHO: World Health Organization
