# Supplementary material for: VIBRA trial – Effect of village-based refill of ART following home-based same-day ART initiation vs clinic-based ART refill on viral suppression among individuals living with HIV: protocol of a cluster-randomized clinical trial in rural Lesotho
Source: Trials. 2019 Aug 22;20:522. doi: 10.1186/s13063-019-3510-5 (PMC6704675; doi:10.1186/s13063-019-3510-5)
Supplement: Supplementary file 1 — GET ON electronic case report form. (PDF 1050 kb) [file 13063_2019_3510_MOESM1_ESM.pdf]

Coversheet for household

Swiss TPH
Swiss Tropical and Public Health Institute
Schweizerisches Tropen- und Public Health-Institut

Name of Data Collector [1]

Other, specify [2]

Date Enrollment/HOSENG [3]

Time of Record [4]

Village/Cluster [5]

District [6]
- ◯ Butha-Buthe
- ◯ Mokhotlong

Corresponding health facility [7]

Study Arm [8]

Household ID [9]

Household occupied? [10]
- ◯ Yes
- ◯ No

Household consent given? [11]
- ◯ Yes
- ◯ No

Reason for refusal [12]

Other, specify [13]

Are there any household members absent today? [14]
- ◯ Yes
- ◯ No

Will any of the absent members return within the next 3 months? [15]
- ◯ Yes
- ◯ No

Are any of these absent members older than 12 years of age? [16]
- ◯ Yes
- ◯ No

Are any of these absent members HIV-negative or unknown HIV status? [17]
- ◯ Yes
- ◯ No

Demographics for individual

Swiss TPH
Swiss Tropical and Public Health Institute
Schweizerisches Tropen- und Public Health-Institut

Household ID [ ] 1

Individual ID [ ] 2

Last name [ ] 3

First name [ ] 4

Who is the main caregiver for the child? [ ] 5

Year of birth (day and month optional) [ ] 6

Is the child an orphan? 7
- ○ Yes, single orphan
- ○ Yes, double orphan
- ○ No

Gender 8
- ○ Female
- ○ Male

Are you pregnant? 9
- ○ Yes
- ○ No

Expected date of delivery (mm/yyyy) [ ] 10

Next scheduled ANC visit? (mm/yyyy)
(Leave blank if no scheduled ANC visit) [ ] 11

Completed years of primary schooling [ ] 12

Completed years of secondary schooling [ ] 13

Completed years of tertiary schooling [ ] 14

Employment status (click all that apply)

- Employed in Lesotho ○ ' 15
- Employed in RSA ○ ' 16
- Self-employed with regular income ○ ' 17
- Younger than school age ○ ' 18
- Student (primary to tertiary) ○ ' 19
- Subsistence farming ○ ' 20
- Housewife ○ ' 21
- No regular income / unemployed ○ ' 22

What is the profession of the individual? [ ] 23

Other, specify [ ] 24

Cell phone

Will individual provide cellphone number?
- ◯ Yes 25
- ◯ No, no phone
- ◯ No, refused to give

Country code [ ] 26

Cellphone number
(please do not include country code or spaces) [ ] 27

Owner of cellphone
- ◯ Participant 28
- ◯ Family member/friend
- ◯ Other

Is the individual absent?
- ◯ Yes 29
- ◯ No

Absent members - individual level

Swiss TPH
Swiss Tropical and Public Health Institute
Schweizerisches Tropen- und Public Health-Institut

Reason for being absent [ ] 1

Specify other reason [ ] 2

When will he/she back? [ ] 3

What is his/her HIV status? [ ] 4

Proof of HIV status or ART drugs given? [ ] 5

OraQuick left for him/her? 6

- ○ Yes
- ○ No, individual <12yrs
- ○ No, household refused OraQuick
- ○ No, absent individual is mentally not able to perform oral HIVST (according to household members)
- ○ No, Other reasons

Other, specify [ ] 7

Please select which health center or health worker the test will be brought back to:

○ ' 9 [ ] 8

○ ' 10 [ ] 11

○ ' 13 [ ] 12

○ Other 14

Other, specify [ ] 15

Data entry for this individual is complete.
Are there any more individuals in this household?

If Yes: Please press F4 and select 'Yes' in the popup
This is the end of the visit. Would you like to move to the next Visit?

If No: Please save the Form and go back to the Home Page

CAGE

Swiss TPH
Swiss Tropical and Public Health Institute
Schweizerisches Tropen- und Public Health-Institut

Do you ever drink alcohol? ◯ Yes ◯ No ◯ Refused to answer 1

How often do you have a drink containing alcohol? 2

Have you ever felt you should cut down on your drinking? 3

Have you ever been annoyed at others for criticising your drinking? 4

Have you ever felt bad or guilty about your drinking? 5

Have you ever taken a drink first thing in the morning to get rid of a hangover or steady your nerves? 6

HIV status

Swiss TPH
Swiss Tropical and Public Health Institute
Schweizerisches Tropen- und Public Health-Institut

Have you ever tested for HIV? 1
- ◯ Yes
- ◯ No
- ◯ I don't know
- ◯ Refused to answer

When was your last HIV test? 2
- ◯ 12 or more months ago
- ◯ less than 12 months ago

What was the result of the last HIV test? [ ] 3

Proof of HIV status provided (bukana)? 4
- ◯ Yes
- ◯ No

Proof of HIV-negative status within last 4 weeks? 5
- ◯ Yes
- ◯ No

Are you on ART (medication against HIV; not from a traditional healer)? [ ] 6

Did they stop ART within the last 4 weeks? 7
- ◯ Yes
- ◯ No

Proof he/she is on ART (bukana or drugs)? 8
- ◯ Yes
- ◯ No

HIV testing

HTC consent given?
- ◯ Yes
- ◯ No, consent refused
- ◯ No, no HIV testing needed, individual can provide proof (bukana/drugs) that he/she is on ART
- ◯ No, no HIV testing needed, individual can provide proof (bukana) of an HIV-positive result
- ◯ No, no HIV testing needed, individual can provide proof (bukana) of an HIV-negative result within the last 4 weeks
- ◯ No, no HIV testing, because child is too young for Determine

Give reason
[ ] 2

Other, specify [ ] 3

OraQuick used for this individiual?
[ ] 4

Other, specify [ ] 5

Please select which health center or health worker the test will be brought back to:
- ◯ ' 6 [ ] 7
- ◯ ' 8 [ ] 9
- ◯ ' 11 [ ] 10
- ◯ Other 12

Other, specify [ ] 13

HIV testing (click all that apply)

Is this the person you test and train on OraQuick?
- ◯ Yes 14
- ◯ No

OraQuick results
- ◯ Positive 15
- ◯ Negative

Determine result
- ◯ Positive 16
- ◯ Negative

UniGold results
- ◯ Positive 17
- ◯ Negative

What was the final result after parallel testing?
- ◯ HIV-positive 18
- ◯ HIV-negative
- ◯ Indeterminate

TB screening

Are you currently on TB treatment? [1]
- ◯ Yes
- ◯ No
- ◯ Refused to answer

When did you start taking TB treatment? (dd/mm/yyyy) [2]

History of TB in the past? [3]

Was it treated? [4]
- ◯ Yes, fully treated (6 months)
- ◯ Yes, partially (less than 6 months)
- ◯ No
- ◯ I don't know

What is his/her HIV status? [5]
- ◯ HIV-positive
- ◯ HIV-negative
- ◯ Unknown
- ◯ Indeterminate

Any signs of TB (in HIV-negative)?

Cough (persistently since 2 weeks) ◯ Yes ◯ No [6]

Lost weight (without trying) of more than 1.5 kg in a month ◯ Yes ◯ No [7]

Fever (persistently since 2 weeks) ◯ Yes ◯ No [8]

Night sweats (persistently since 2 weeks) ◯ Yes ◯ No [9]

Child fails to thrive/faltering growth or sign of severe malnutrition ◯ Yes ◯ No [10]

Child has been in contact with someone with TB disease ◯ Yes ◯ No [11]

Any signs of TB (in HIV-positive)?

Cough (of any duration) ◯ Yes ◯ No [12]

Lost weight (without trying) of more than 1.5 kg in a month ◯ Yes ◯ No [13]

Fever (of any duration) ◯ Yes ◯ No 14

Night sweats (of any duration) ◯ Yes ◯ No 15

Child fails to thrive/faltering growth or sign of severe malnutrition ◯ Yes ◯ No 16

Child has been in contact with someone with TB disease ◯ Yes ◯ No 17

[ ] 18

On-spot sputum collected? ◯ Yes ◯ No 19

Reason sputum not collected [ ] 20

Other, specify [ ] 21

Condoms

Swiss TPH
Swiss Tropical and Public Health Institute
Schweizerisches Tropen- und Public Health-Institut

Condoms provided? [1]

- ○ Yes
- ○ No
- ○ Refused

VMMC

Swiss TPH
Swiss Tropical and Public Health Institute
Schweizerisches Tropen- und Public Health-Institut

VMMC referral accepted?

- ◯ Yes [1]
- ◯ No

VIBRA eligibility

Swiss TPH
Swiss Tropical and Public Health Institute
Schweizerisches Tropen- und Public Health-Institut

Eligibility criteria

Has a body weight of <35 kg? ◯ Yes ◯ No [1]

Currently in care for high blood pressure (hypertension) or high blood sugar (diabetes)? ◯ Yes ◯ No [2]

HIV-positive individual is physically, mentally, or emotionally not able to participate in the study, in the opinion of the investigators or study staff ◯ Yes ◯ No [3]

HIV-positive individual wishes to get care outside the study districts ◯ Yes ◯ No [4]

Eligible for VIBRA [5]

Nurse entering data [6]

Specify the name of the other nurse [7]

VIBRA demographics

Swiss TPH
Swiss Tropical and Public Health Institute
Schweizerisches Tropen- und Public Health-Institut

Was this person re-tested for HIV?
- ◯ Yes [1]
- ◯ No, they are known HIV+ defaulters, i.e. have already been re-tested.

How do you travel to the nearest health facility?

Taxi ◯ [2]

Walk ◯ [3]

Horse/donkey ◯ [4]

Bicycle ◯ [5]

Own car ◯ [6]

Driven by friend/family member ◯ [7]

How long does it usually take you to travel one-way to the nearest health facility? (minutes) [ ] [8]

How much does it usually cost you to travel one way to the nearest health facility? (If child, include cost of caregiver) (Loti/Rand) [ ] [9]

Would you lose any money during the time you need to access the health facility? [10]
- ◯ Yes
- ◯ No

How much money would you lose? (Loti/Rand) [ ] [11]

How many children do you have? [ ] [12]

Would you need to pay a caregiver for your children (or other people you take care of) while accessing the health facility? [13]
- ◯ Yes
- ◯ No
- ◯ I don't have children/persons to take care of

Will you tell a close person (friend or family member) that you tested HIV-positive?
(If child, will somebody else than the caregiver know?) [ ] [14]

Do you live with an HIV-positive household-member? [ ] [15]

Do you have regular sexual partner(s)? [ ] [16]

Do you know if any of your sexual partner(s) is also HIV-positive?

17

HIV knowledge

Swiss TPH
Swiss Tropical and Public Health Institute
Schweizerisches Tropen- und Public Health-Institut

Is HIV spread by kissing? 1

Can a person get HIV by sharing kitchens or bathrooms with someone who has HIV? 2

Can you get HIV by touching someone who has HIV? 3

Can men give HIV to women? 4

Can women give HIV to men? 5

Must a person have many different partners to get HIV? 6

Does washing after sex help protect against HIV? 7

Can a pregnant woman give HIV to her baby? 8

Can a person get rid of HIV by having sex with a virgin? 9

Is there a cure for HIV? 10

Medical history

Swiss TPH
Swiss Tropical and Public Health Institute
Schweizerisches Tropen- und Public Health-Institut

Have you ever been on ART? [ ] 1

Do you remember the dates you previously took ART? ◯ Yes ◯ No 2

When did you start taking ART? (mm/yyyy) [ ] 3

When did you stop taking ART? (mm/yyyy) [ ] 4

Why did you stop taking ART? [ ] 5

Other, specify [ ] 6

Do you remember the ART regimen? ◯ Yes ◯ No 7

What was the ART regimen? [ ] 8

If other please specify [ ] 9

Have you ever been on PMTCT/PEP/PrEP? [ ] 10

Do you remember when? ◯ Yes ◯ No 11

When did you start taking PMTCT/PEP/PrEP? (mm/yyyy) [ ] 12

When did you stop taking PMTCT/PEP/PrEP? (mm/yyyy) [ ] 13

Do you remember the PMTCT/PEP/PrEP regimen? ◯ Yes ◯ No 14

What was the PMTCT/PEP/PrEP regimen? [ ] 15

Have you been told that you have other diseases or health problems (besides HIV)? [ ] 16

What other diseases or conditions do you have? [ ] 17

Are you currently taking any medication? [ ] 18

Please specify [ ] 19

Do you currently smoke cigarettes/nicotin? [20]

- ◯ Yes
- ◯ No
- ◯ Refused to answer

Do you currently smoke Dagga? [21]

- ◯ Yes
- ◯ No
- ◯ Refused to answer

Physical exam

Swiss TPH
Swiss Tropical and Public Health Institute
Schweizerisches Tropen- und Public Health-Institut

Body weight (kilograms) [ ] 1

Does the patient report any diarrhea? ◯ Yes ◯ No 2

Date of onset (dd/mm/yyyy) [ ] 3

Frequency (Episodes Per Day) [ ] 4

Color [ ] 5

After examination, do you suggest referral with further consultation before starting ART? ◯ Yes ◯ No 6

Does the patient report any headache? ◯ Yes ◯ No 7

Date of onset (dd/mm/yyyy) [ ] 8

Location of headache [ ] 9

Pain intensity (1-10) [ ] 10

After examination, do you suggest referral with further consultation before starting ART? ◯ Yes ◯ No 11

Does the patient report any other symptoms (other than diarrhea or headache)? ◯ Yes ◯ No 12

Other, specify [ ] 13

After examination, do you suggest referral with further consultation before starting ART? ◯ Yes ◯ No 14

Does the patient present oral thrush? ◯ Yes ◯ No 15

Does the patient present any abnormal skin lesions (incl. lips, scalp)? ◯ Yes ◯ No 16

Please specify [ ] 17

Does the patient present any lymphadenopathie? ◯ Yes ◯ No 18

Specify location [ ] 19

Does the patient present any other condition (other than oral thrush, abnormal skin lesion, lymphadenopathie)?

- ◯ Yes [20]
- ◯ No

Please specify [21]

Do(es) the condition(s) suggest referral with further consultation before starting ART?

- ◯ Yes [22]
- ◯ No

WHO stage

- ◯ 1 [23]
- ◯ 2
- ◯ 3
- ◯ 4

Laboratory work

Swiss TPH
Swiss Tropical and Public Health Institute
Schweizerisches Tropen- und Public Health-Institut

Haemoglobin done? ◯ Yes ◯ No 1

Haemoglobin result (g/dl) [ ] 2

If not done: specify reason [ ] 3

Other reasons [ ] 4

Creatinine done? ◯ Yes ◯ No 5

Creatinine result (micromol/l) [ ] 6

If not done: specify reason [ ] 7

Other reasons [ ] 8

eGFR (mL/min) [ ] 9

CD4-count done? ◯ Yes ◯ No 10

CD4 result (cells/microL) [ ] 11

If not done: specify reason [ ] 12

Other reasons [ ] 13

CrAg screening result ◯ Positive ◯ Negative ◯ Not done 14

If CrAg not done: specify reason [ ] 15

If CrAg not done for other reasons, specify [ ] 16

Venous blood has to be taken. Did you take venous blood? ◯ Yes ◯ No 17

If no, give reason [ ] 18

Other, specify [ ] 19

Does the patient need to be referred to the health facility for any clinical (medical history, physical exam) or laboratory result reason?

- ◯ Yes [20]
- ◯ No

Specify reason [21]

Readiness

What do you think: How would you remember to take your medication every day?

Mobile phone reminder ◯ ' 1

Alarm on a clock or watch ◯ ' 2

Ask someone to remind me ◯ ' 3

Use a calendar or diary ◯ ' 4

Take my tablets at the same time as I do something else every day (like brushing my teeth) ◯ ' 5

No reminders, I'll just remember ◯ ' 6

Other reminder ◯ ' 7

if other reminder: specify [ ] 8

Is there anything that would stop you from taking your tablets every day? ◯ Yes ◯ No 9

If yes: specify [ ] 10

If you should start ART today, how ready are you? 11
◯ Ready today
◯ Thinking about starting in the coming days
◯ Not ready

Indicate date of suggested ART start [ ] 12

Did the patient raise any issues or serious concerns that lead you to think that ART initiation should be delayed? ◯ Yes ◯ No 13

If yes: specify [ ] 14

Does the patient need to be referred to the health facility due to concerns with readiness? ◯ Yes ◯ No 15

Adherence counseling

Swiss TPH
Swiss Tropical and Public Health Institute
Schweizerisches Tropen- und Public Health-Institut

Adherence/education session delivered? ◯ Yes ◯ No 1

If no: give reason [ ] 2

If other reasons: specify [ ] 3

Adherence leaflet left? ◯ Yes ◯ No 4

If no: give reason [ ] 5

If other reasons: specify [ ] 6

VIBRA acceptance

Swiss TPH
Swiss Tropical and Public Health Institute
Schweizerisches Tropen- und Public Health-Institut

Do you want to get your ARV refill through your VHW? ○ Yes ○ No 1

If no: specify [ ] 2

What is the name of the responsible VHW?

○ ' 4 [ ] 3

○ ' 6 [ ] 5

Cellphone number of the VHW given to participant? ○ Yes ○ No 7

If no: give reason [ ] 8

Do you want to receive SMS (as reminders and lab result info) for the follow-up period? ○ Yes ○ No 9

If no: specify why not [ ] 10

Date of next follow-up visit (within 12-16 days) at the VHW? (dd/mm/yyyy) [ ] 11

ART dispensing

Swiss TPH
Swiss Tropical and Public Health Institute
Schweizerisches Tropen- und Public Health-Institut

Which ART regimen provided? [1]
- ◯ TDF/3TC/EFV
- ◯ ABC/3TC/EFV
- ◯ AZT/3TC/EFV
- ◯ Other

If other: specify [2] ______

Cotrimoxazole (CTX) given? [3]
- ◯ Yes
- ◯ No, because CD4 >=350 cells/microL
- ◯ No, because out of stock
- ◯ No, because patient refused
- ◯ No, other reason

If other reason: specify [4] ______

Do you want to receive SMS appointment reminders for the follow-up visits at the health facility? [5]
- ◯ Yes
- ◯ No

if no: specify why not [6] ______

Date of next follow-up visit (within 12-16 days) at the health facility? [7] ______

Work-up health facility

Swiss TPH
Swiss Tropical and Public Health Institute
Schweizerisches Tropen- und Public Health-Institut

pre-ART number (xx-xx/xxxxx) [1] - [2] / [3]

ART number (x-xx/xxxxx) [4] - [5] / [6]

Follow-up

Swiss TPH
Swiss Tropical and Public Health Institute
Schweizerisches Tropen- und Public Health-Institut

Date today (mm/dd/yyyy) [1]

Subject ID [2]

ART number [3]

Currently pregnant? [4]
- Yes
- No

Date of last menses? (mm/yyyy) [5]

Pregnant at last visit? [6]
- Yes
- No

Please specify outcome [7]

Does the patient report a cough? [8]
- Yes
- No

Days since onset [9]

Is the cough productive? [10]
- Yes
- No

Does the patient report weight loss (without trying)? [11]
- Yes
- No

Does the patient report drenching/soaking sweats at night? [12]
- Yes
- No

Does the patient report having fevers? [13]
- Yes
- No

Does the patient report any headache, dizziness, disturbing dreams or a confused behaviour? [14]
- Yes
- No

Days since onset? [15]

Does the patient report any abdominal pain or nausea? [16]
- Yes
- No

Decrease of food intake? [17]
- Yes
- No

Does the patient report any vomiting? [18]
- Yes
- No

Days since onset [19]

Frequency (episodes per day) [ ] 20

With blood? ◯ Yes ◯ No 21

Does the patient report any diarrhea? ◯ Yes ◯ No 22

Days since onset [ ] 23

Frequency (episodes per day) [ ] 24

With blood? ◯ Yes ◯ No 25

Does the patient report any other symptoms? ◯ Yes ◯ No 26

Specify [ ] 27

Does the patient present oral thrush? ◯ Yes ◯ No 28

Does the patient present any skin rash? ◯ Yes ◯ No 29

itchy? ◯ Yes ◯ No 30

painful? ◯ Yes ◯ No 31

location [ ] 32

Does the patient present any lymphadenopathie? ◯ Yes ◯ No 33

Location [ ] 34

Does the patient present any other new condition? ◯ Yes ◯ No 35

specify [ ] 36

Did the patient miss any dose of ART on any day during the last month (30 days)? ◯ Yes ◯ No 37

If yes, how many days did he/she miss? [ ] 38

Specify reason for missing doses [ ] 39

Did the patient miss doses on two or more following days during the last month?
◯ Yes
◯ No
40

Did the patient go to any other health facility since last visit?
◯ Yes
◯ No
41

Clinic name ____ 42

Reason ____ 43

Did the patient disclose his/her HIV-status to others?
◯ Yes
◯ No
◯ Refused to answer
44

According to you: How well is the patient coping with the disease? ____ 45

ART regimen modified today?
◯ Yes
◯ No
46

Give reason ____ 47

Other, specify ____ 48

ART regimen provided today ____ 49

Other, specify ____ 50

Isoniazid Preventive Therapy (IPT) given today?
◯ Yes
◯ No
51

Co-trimoxazole (CTX) given today?
◯ Yes
◯ No
52

Any other drugs given?
◯ Yes
◯ No
53

If yes: specify (name, and other info if available ____ 54

Next appointment date for follow-up visit (dd/mm/yyyy) ____ 55

Name of person that performed the follow-up ____ 56

Blood drawn today?
◯ Yes
◯ No
57

Date of (first) blood analysis (dd/mm/yyyy) ____ 58

ALT, U/l ____ 59

AST, U/l ____ 60

Hemoglobin, g/dl [ ] 61

CD4, cells/microL [ ] 62

Creatinine, micromol/L [ ] 63

VL taken? 64
- ◯ Yes
- ◯ No

Date of VL analysis (dd/mm/yyyy) [ ] 65

VL: below detectable limit (<20 copies/mL) 66
- ◯ Yes
- ◯ No

VL, c/mL [ ] 67

Any other tests performed today? 68
- ◯ Yes
- ◯ No

If yes: specify [ ] 69

Adverse events

Swiss TPH
Swiss Tropical and Public Health Institute
Schweizerisches Tropen- und Public Health-Institut

Subject ID [1]

ART number [2]

Specify Adverse Event [3]

Serious Adverse Event? [4]

Start Date (dd/mm/yyyy) [5]

Severity [6]
- ◯ Mild
- ◯ Moderate
- ◯ Severe

Current ART regimen [7]

Other, specify [8]

Relationship to current ART [9]
- ◯ Clearly not related to the intervention (current ART)
- ◯ May be related to intervention (current ART)
- ◯ Clearly related to intervention (current ART)

Stop Date (dd/mm/yyyy) [10]

Outcome of AE [11]

Initials of person filling in the form [12]

Tracing form

Swiss TPH
Swiss Tropical and Public Health Institute
Schweizerisches Tropen- und Public Health-Institut

Individual ID [1]

ART number [2]

Date of information or tracing attempt (dd/mm/yyyy) [3]

How was the information obtained? [4]

Outcome of tracing? [5]

Date of death (dd/mm/yyyy) [6]

Cause of death [7]

Name of clinic [8]

Date of transfer (dd/mm/yyyy) [9]

Specify reason for stopping ART [10]
